# Vancomycin-Resistant Enterococci and Its Associated Risk Factors among HIV-Positive and -Negative Clients Attending Dessie Referral Hospital, Northeast Ethiopia

**DOI:** 10.1155/2018/4753460

**Published:** 2018-07-18

**Authors:** Seid Ali, Martha Alemayehu, Mulat Dagnew, Teklay Gebrecherkos

**Affiliations:** ^1^Microbiology Laboratory Unit, Dessie Referral Hospital, Amhara Region, Dessie, Ethiopia; ^2^Department of Medical Microbiology, School of Biomedical and Laboratory Sciences, College of Medicine and Health Sciences, University of Gondar, Gondar, Ethiopia

## Abstract

**Background:**

Enterococci are becoming the most important public health concern and emerging as multidrug-resistant organisms around the world including Africa particularly in Ethiopia where there is a lack of availability of effective antimicrobial drugs. However, there is a paucity of data on the prevalence and associated risk factors of vancomycin-resistant enterococci in Ethiopia.

**Objective:**

This study was aimed to assess the prevalence of vancomycin-resistant enterococci and its associated risk factors among HIV-positive and -negative clients.

**Methods:**

A comparative cross-sectional study was conducted from February to May, 2017, on 300 participants at Dessie Referral Hospital. Data were gathered using a pretested structured questionnaire, stool samples were collected and inoculated on to bile esculin agar, and presumptive colonies were inoculated in brain-heart infusion broth containing 6.5% NaCl for selective identification of enterococci. Antibiotic susceptibility tests were done using the Kirby–Bauer disk diffusion method. Data were analyzed using SPSS version 22 software package.

**Results:**

A total of 300 study participants were enrolled in this study, of which 57.7% were females with a mean age of 34.4, a range of 19–73 years. The overall prevalence of enterococci was 37.3%. The prevalence of VRE was 6.3%. From all isolates, the prevalence of VRE among HIV-positive and -negative clients was 5.9% and 7.4%, respectively. Resistance gentamicin, ampicillin, penicillin, and erythromycin was 37.5%, 34.8%, 34.8%, and 22.3%, respectively. Prevalence of multidrug resistance was (29.5%). Being low in hemoglobin content was significantly associated with VRE.

**Conclusion:**

The high prevalence of VRE and multidrug-resistant enterococci in this study signals the emergence of VRE. Detection of VRE in this study indicates decreased antibiotic treatment options of multidrug-resistant enterococci. Therefore, there should be a need to perform continuous surveillance, rational use of antibiotics, and more detailed study using phenotypic and genotypic methods.

## 1. Introduction

Enterococci are Gram-positive bacteria arranged in pairs and short chains, and they are part of facultative anaerobic normal flora of both humans and animals. They are normally found in the oropharynx, vagina, urethra, and skin of healthy persons [1]. Previously, it is considered as bacteria of minimal clinical impact, particularly *Enterococcus faecium* and *Enterococcus faecalis*, and has now emerged as one of the major causes of human clinical infections [2]. Enterococci are productive colonizers, with genome flexibility and a tendency for persistency in hospital environments. It causes urinary tract infections mainly followed by intra-abdominal and pelvic infections. They also cause surgical wound infections, bacteremia, endocarditis, neonatal sepsis, and rarely meningitis [3].

The growing evidences show that enterococci are among the third and fourth leading cause of nosocomial infections worldwide [4]. Studies indicate that, in hospitals, it is the second most common cause of catheter-associated bloodstream infections [5]. For instance in the United States, 18% of all bloodstream infections were due to enterococci, ranking as second from overall infections [6]. In Africa, enterococci prevalence was documented in different clinical samples ranging from 5.7% to 88.9% [7–9].

Moreover, enterococci acquire resistance to currently available drugs either by mutation or receipt of foreign genetic materials through the transfer of plasmids and transposons, or they have a selective pressure over other microorganisms of the intestinal flora, allowing for overgrowth of intrinsic vancomycin-resistant enterococci (VRE). Despite the fact that the antibiotics are no longer effective due to the emergence of VRE is becoming a grave public health concern especially in developing countries where there is a lack of availability of effective antibiotics [10]. Furthermore, VRE have been emerging and posing a therapeutic challenge to physicians due to the ease of acquiring and transferring antimicrobial-resistant genes and presence of different selection pressures for VRE proliferation and rapid expansion of resistant populations.

Evidences showed that previous administration of antibiotic treatment, concurrent infections, surgery, catheterizations, duration of hospital stay, presence of pervious hospitalization, and underlying diseases like cancer, HIV, and diabetics are among the risk factors associated with the spread of enterococci infections [4, 7, 8].

There were published studies concerning the burden of enterococci, drug-resistant enterococci, and VRE worldwide and in Africa. However, little is known about the prevalence of VRE in the susceptible group of individuals like HIV-infected patients in Ethiopia. Thus, the aim of this study was to determine the prevalence of enterococci, VRE, and other antimicrobial-resistant enterococci among HIV-positive and -negative clients at Dessie Referral Hospital.

## 2. Materials and Methods

### 2.1. Study Area, Design, and Period

A hospital-based comparative cross-sectional study was conducted from February to May, 2017, at Dessie Referral Hospital, South Wollo zone of Amhara regional state, Northeast Ethiopia. This hospital is found in Dessie town with a distance of 400 km from the capital (Addis Ababa) and 471 km far from Bahir Dar, which is the capital city of the Amhara regional state. According to 2007 population and housing census of Ethiopia, the town had a total population of 151,094; among these, 72,891 were males, and 78,203 were females.

In Dessie town, there are one referral hospital, three private general hospitals, five health centers, eight private higher clinics, and one regional health and research laboratory. Dessie Referral Hospital provides emergency, ART services, chronic care, surgical, dental, medical, pediatric, gynecologic, obstetric, and other services for more than 4 million clients annually.

### 2.2. Study Population, Sample Size, and Sampling Procedure

HIV-positive patients from ART clinic and healthy blood donors attending Dessie Referral Hospital during the study period were enrolled in this study. The sample size was determined by using EPI INFO version 7 in double population proportion. Since there was no previous study done in this area, 50% prevalence was given to HIV-negative controls, while the proportion given for HIV-positive cases was 70%. This was depending on an assumption that HIV-positive clients have 20% more VRE carriage rate than HIV-negative clients [7]. EpiInfo soft was used to determine the sample size by considering power 80% and confidence interval at 95% for the two sample sizes with a 1 : 1 ratio; the initial sample size was 206, by considering 10% nonresponse rate with a total of 300 study participants (150 from HIV positives and 150 from HIV negatives). A systematic random sampling technique was used to select study participants.

### 2.3. Definition of Terms

- Body mass index (BMI): BMI is a simple index of weight-for-height that is commonly used and is defined as the weight in kilograms divided by the square of the height in meters (kg/m^2^). It is used to assess the physical and nutritional status.
- Hospitalization: Admission and waiting in the hospital for more than 48 hours or visiting health institutions repeatedly.
- MDR: Bacteria that is nonsusceptible to ≥3 antimicrobial class.

## 3. Data Collection and Laboratory Methods

### 3.1. Data Collection

Data were collected using a short interview guided by a pretested structured questionnaire consisting of the client's information like demographic characteristics, dates of recent hospitalization, immune status, duration of hospitalization before the onset of the sample collection, and invasive procedures which was obtained for each study subject either from the clinical record or from the interview. Five milliliters of blood sample was collected by experienced laboratory technologists. The HIV status of the clients was determined using rapid test kits following the current Ethiopian algorithm (for healthy blood donors), and CD4 count, WHO staging of HIV, and ART linkage (for HIV positives) were collected from their charts/request papers. Data were collected by trained nurses with previous experience and speaking local language.

### 3.2. Stool Sample Collection

Each patient was instructed how to collect stool specimens, and about 5 mg of the fecal specimen was collected in a sterile wide-mouth screw-capped container from each patient and labeled with the unique sample number, date, and time of collection. Immediately, it was delivered to bacteriology laboratory (Dessie Regional Health and Research Laboratory) for culturing within 30 minutes. The collected samples were kept in the refrigerator for 1-2 hours if unprocessed immediately [11].

### 3.3. Sample Processing and Culture Identification

The media were prepared as per the manufacturers' instructions. The stool sample collected from each client was streaked on sterile bile esculin agar media (BEA) (OXOID, Hardy Diagnostics, Santa Maria, CA, USA) and incubated for 24–48 hours at 37°C. Plates were observed for appearance of characteristic colonies of growth and blackening. Typical characteristic colonies were selected randomly for characterization and presumptive identification of enterococci. Each isolate was also assessed using Gram staining, catalase test, and its growth in 6.5% NaCl broth. Presumptive pure colonies were picked and inoculated into brain-heart infusion (BHI) broth and incubated at 45°C for 24 hours, and growth in the medium was assessed by its turbidity. An isolate fulfilling the above criteria was assumed to be *Enterococcus* species [12].

### 3.4. Antimicrobial Susceptibility Testing

The antimicrobial susceptibility testing of the isolates was performed on Mueller–Hinton agar (OXOID, UK) by the Kirby–Bauer disk diffusion technique as modified by the Clinical and Laboratory Standard Institute in 2017 [13]. After a pure culture was obtained, a loop full of bacteria was taken from a colony and was transferred to a tube containing 5 ml of sterile normal saline (0.85% NaCl) and mixed gently until it formed a homogenous suspension. The turbidity of the suspension was determined in comparison with 0.5 McFarland standards. A sterile swab was dipped into the suspension, and excess suspension was removed by pressing the swab against the wall of the tube. The swab was used to distribute the bacteria suspension evenly over the entire surface of MHA.

The inoculated plates were left at room temperature for 3–5 minutes until it dried. The antimicrobial impregnated disks were placed with sterile forceps on the agar surface at least 24 mm away from each other to avoid the overlapping zone of inhibition. After placing the disk, the plate was allowed to stand for 30 minutes to dissolve the antibiotic in the media. Then, the plates were inverted and incubated at 37°C for 18–24 hrs, and the diameter of the zone of inhibition was determined using a ruler. Susceptibility pattern was categorized as sensitive, intermediate, and resistant by comparison of the zone of inhibition with a standard indicated in the manufacturer's guide.

The isolated enterococci were tested against penicillin (10 IU), ampicillin (10 *μ*g), vancomycin (30 *μ*g), gentamicin (30 *μ*g), ciprofloxacin (5 *μ*g), erythromycin (15 *μ*g), and chloramphenicol (30 *μ*g).

Then, the plates were incubated at 37°C for 24 hours, and the results were interpreted according to the most recent version of Clinical Laboratory Standard Institute (CLSI 2017) [13].

### 3.5. Data Quality Assurance

The standard reference bacterial strains such as the reference strain of *S. aureus* ATCC25923 and *E. faecalis* ATCC51299 were used as negative and positive controls, respectively, and tested weekly as controls on the biochemical tests and agar plates including Mueller–Hinton agar with antimicrobial discs to ensure testing performance of the potency of antimicrobial discs. Sterility of the culture media was tested by incubating 5% of the batch at 35–37°C overnight and was evaluated for possible contamination. In addition, the whole procedures and result interpretation were cross-checked by senior laboratory professionals of the Dessie regional health and research laboratory.

### 3.6. Data Processing and Analysis

Data were checked for completeness, cleaned manually, entered, and analyzed using the Statistical Package for Social Science (SPSS) version 22. Frequencies and cross tabulations were used to summarize descriptive statistics. The association of dependent and independent variables was measured by using bivariate and multivariate analysis (logistic regression). The strength of the association was measured by the odds ratio. At 95% of confidence interval, a *p* value < 0.05 was considered as statistically significant.

### 3.7. Ethical Consideration

The study was conducted after getting ethical approval and support letter from the ethical Committee of School of Biomedical and Laboratory Sciences, College of Medicine and Health Sciences, University of Gondar. Written informed consent was obtained from each of the study participants. For each confirmed resistant case, the responsible clinicians of the patient were informed about the results of the clients using letters that contain results of all clients.

## 4. Results

### 4.1. Sociodemographic Characteristics

A total of 300 study participants were enrolled in this study. The mean age and standard deviation was 34.4 ± 10.01, and age range was from 19 to 73 years. Of the 300 study participants, 150 were HIV positive (with mean age and standard deviation of 38.50 ± 10.95, age range 19–73) and 150 were HIV negative (with mean and standard deviation of 30.35 ± 6.91, age range 19–56). Majority of the study participants were females, 173/300 (57.3%), and their educational level was mainly primary education, 89/300 (29.7%). In addition, most of the study participants were married, and 166/300 (55.3%) and 255/300 (85%) participants were urban dwellers (Table 1).

### 4.2. Prevalence of Enterococci and VRE among HIV-Positive and HIV-Negative Clients

**Figure 1:**
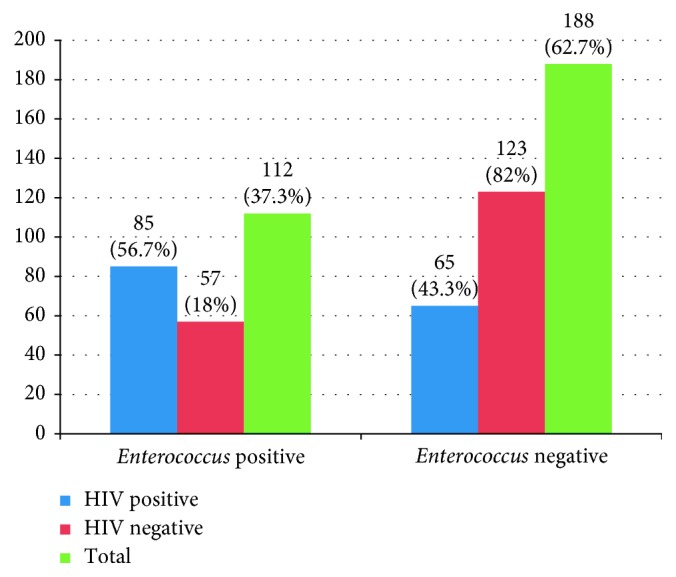
Prevalence of enterococci among HIV-positive and -negative clients at Dessie Referral Hospital, Northeast Ethiopia, 2017.

From the total of 300 study participants, colonization of enterococci species was seen on 112 (37.33%) stool specimens (95% CI = 31.2–42.8%). Of this, 85 (56.7%) (95% CI = 48–64%) of HIV-positive clients and 27 (18%) (95% CI = 11.7–24) of HIV-negative clients were colonized with isolates of enterococci species (Figure 1).

From all 112 isolates, 7 (6.3%) were vancomycin-resistant enterococci (VRE) (95% CI = 1.8–10.7). Of all enterococci isolates, VRE among HIV-positive and -negative clients were 5/85 (5.9%) (CI = 2.4–10.7) and 2/27 (7.4%) (95% CI = 0–18.5), respectively.

### 4.3. Antimicrobial-Resistant Patterns of Enterococci to Other Antibiotics

The resistance pattern of enterococci for each ampicillin and penicillin was 39 (34.8%); of these, 32 (37.6%) and 7 (25.9%) isolates were resistant to HIV-positive and HIV-negative clients, respectively. Forty-two (37.5%) isolates were resistant to gentamicin, of which 38 (44.7%) were from HIV-positive clients and 4 (14.8%) were from HIV-negative clients. Twenty-five (22.3%) isolates were resistant to erythromycin; all of the isolates (29.4%) were from HIV-positive clients only.

Statistically significant difference of resistant patterns was seen on gentamicin and erythromycin between isolates among HIV-positive and HIV-negative clients (*p* < 0.05). However, no statistically significant difference was observed in resistant patterns for ampicillin, penicillin, ciprofloxacin, chloramphenicol, and vancomycin among HIV-positive and HIV-negative clients (*p* > 0.05) (Table 2).

From the total enterococci isolates, 60 (53.6%) were resistant to one or more antibiotics; of those isolates, 21 (18.8%), 14 (12.5%), 20 (17.9%), and 5 (4.5%) were resistant to one, two, three, and four antibiotics, respectively. In this study, the overall prevalence of multiple drug resistance (MDR) enterococci was found to be 25/112 (22.3%) (Table 3).

In this study, the most sensitive antibiotic for enterococci isolates was vancomycin (93.7%), while ciprofloxacin and erythromycin were 100% susceptible in HIV-negative individuals. General susceptibility pattern for all antibiotics was shown in (Table 4).

### 4.4. Factors Associated with the Emergence of Vancomycin-Resistant Enterococci

Multivariate analysis was done on those variables that were in significant association with bivariate analysis and *n* = 27 less than 0.2. Accordingly, being low in hemoglobin content was significantly associated with vancomycin-resistant enterococci ((AOR = 19.18, 95% CI = 1.08–89.36), *P* value = 0.04). Clients with low-hemoglobin level were 19.2 times more affected by VRE than normal ones; however, other sociodemographic characteristics and risk factors were not statistically significant with VRE in multivariate analysis (Table 5).

## 5. Discussion

Enterococci are productive colonizers with a tendency for persistency in hospital environments. Likewise, VRE have been emerging and posing a therapeutic challenge to physicians due to the ease of acquiring and transferring antimicrobial-resistant genes [14].

The overall prevalence of enterococci in this study was found to be 37.33%. This finding is comparable with the previous report of India, which was reported to be 39.3% (19), and Shiraz, Iran, 39.5% [15]. However, it is higher than the previous results reported from Nigeria, 5.9% [16], and Gondar, Ethiopia, 6.2% [8]. The difference might be due to the variation of the sample type, which were clinical samples including blood, urine, wound swabs, and sputum in the previous study in Gondar, but the stool sample in the current study, in fact as enterococci are common normal flora of the gastrointestinal tract and undergone selective pressure over other normal flora in immune compromised individuals at the time of antibiotic treatment, their colonization has increased gradually.

Furthermore, the clients in the present study were HIV-positive cases, using repeated antibiotics and more hospitalized, while in the previous study, the clients were more of outpatients, could not use antibiotics regularly, and their hospitalization was not as much to HIV clients. It is evidenced that, in the health care facilities, enterococci may acquire resistance to all currently available antibiotics either by mutation or receipt of foreign genetic materials through the transfer of plasmids and transposons, or they have a selective pressure over other microorganisms of the intestinal flora, allowing for overgrowth of VRE, as it is intrinsically resistant to several antibiotics [10].

On the other hand, the present study showed that the prevalence of enterococci in HIV-positive patients was 56.7%; this is comparable with the result reported from Italy, 62% [17], and the Netherlands, 49% [18], on hospitalized patients. However, this result is lower than that of the previous report on hospitalized patients from Jimma, Ethiopia, 76% [9]. This discrepancy could be due to the difference in geographical location, and the method used in the previous study used the advanced molecular technique.

However, the prevalence of enterococci in HIV-negative controls was found to be 18%, and this is consistence with the result reported from USA, 18% [6]. However, it is lower than that of the previous finding of Egypt, 75% [16]. This might be due to difference in geographical location, and study subjects in the previous study were pediatrics/oncology patients.

The overall prevalence of VRE in this study was found to be 6.3%. This finding is in agreement with reports from Egypt, 6.8% [19], Nigeria, 5.9% [16], and Jimma, Ethiopia, 5% [9]. However, this result is lower than from the previous findings of Benin, 67.5% [20], Iran, 24.8% [21], and Brazil, 23.4% [22]. The lower prevalence of VRE in this study might be due to the study design and study participants (pediatric age and intensive care unit) in the previous study and the methods used for detection of vancomycin-resistant enterococci isolates.

This is true that VRE are circulating in the hospital settings and can be disseminated to the environment in the community. Vancomycin-resistant genes are easily transmitted through transposons which contain van genes and have ability to carry and transmit transferable genetic elements. In addition, in recent years, these genes have been transferring resistant genes to methicillin-resistant *Staphylococcus aureus* to form VRSA, thereby causing failure to treatment [23, 24]. On the contrary, the present study was higher than previous reports conducted from Saudi Arabia, 1.2% [25]. This difference could be because of small sample size, variation of study period, and absence of environmental contamination in previous studies, and clinical samples such as blood, pus, and urine were tested in Saudi Arabia.

The present finding showed that, of the 112 enterococci isolates, the prevalence of VRE among HIV-positive clients was 5.9%, which is in consistence with research done on HIV-positive clients from Gondar, Ethiopia, 7.8% [7]. However, the prevalence of VRE in this study is higher than that reported from California, 0% [26]. The higher prevalence of VRE among HIV positives might be due to repeated exposure to different antibiotics and immune suppression which are important risk factors for colonization or infection with VRE. This variation might also be due to individuals having high-animal contact in Ethiopia; this is evidenced from high prevalence of VRE (100%) in chickens and cattle, which was reported by Bekele et al. from Addis Ababa, Ethiopia [27].

In this study, enterococci tested against commonly used antibiotics showed that vancomycin was highly susceptible than other tested antibiotics. This result is in line with the study conducted in Gondar, Ethiopia, with a susceptibility of vancomycin greater than ampicillin, penicillin, ciprofloxacin, and chloramphenicol [8]. In this study, prevalence of penicillin and ampicillin resistant patterns was 34.8% each; this is in consistence with reports from Egypt, 44.4% for penicillin [19], and Jimma, Ethiopia, 36% for ampicillin [7]. This finding is lower than previous reports of the study from Iran, 72.7% and 69.7% for penicillin and ampicillin, respectively [28]. This variation might be due to the characteristics of the study participants; the study subjects in the previous studies were patients who underwent hemodialysis, who were more intensive, hospitalized, and immune compromised than the clients in the present study, who were HIV-positive clients with no patients from ICU.

On the contrary, the prevalence of gentamicin resistance was 37.5%, and this finding is more likely agreed with result reported from Turkey, 35.7% [29], Egypt, 35.5% [30], and Jimma, Ethiopia, 34.2% [7]. However, it is lower than that reported from Iran, 86% [31]. This might be due to the method used different from the present study; in the previous study, the method used molecular system, which is more sensitive and specific to detect VRE in the gene level.

The prevalence of chloramphenicol in the present study was 8.9%. This finding is more likely comparable with the result reported from Brazil, 10.9% [22]. Although, it is lower than the report of Egypt, 31.6% [30], this difference might be due to study participants and sample type; the sample types in the previous study were various clinical samples including blood, urine, wound swabs, and sputum, and study participants were from pediatric ICU oncology/hematology patients.

From the total of 112 (37.4%) enterococci isolates identified, 25 (29.5%) were multiple drug resistance (MDR), this is comparable with the study done in Gondar, Ethiopia, 33.3% [7], and Addis Ababa, Ethiopia, 29% [32]. However, this finding is less than that reported from Jimma, Ethiopia, 89.5% [9]. This might be due to variation in the study subjects and the method of susceptibility testing. The method used in Jimma, Ethiopia, was minimum inhibitory concentrations (MIC) by using E-test strips for the determination of vancomycin.

Among sociodemographic characteristics in the present study, the prevalence of VRE was high among females (85.7%) than males (14.3%). This was evidenced from various epidemiological studies which showed that prevalence of VRE was more common in females than males of their counterparts, which is in agreement with two reports from Ethiopia [7, 9]. This is because of the fact enterococci carriage rate and VRE are more common on females than males.

The current finding showed that being HIV positive was not significantly associated (*p*=0.78) with the occurrence of VRE; however, there are reports showing that enterococci and VRE have been more common in immune compromised clients in hospital settings, especially in HIV-positive patients [33]. The reason could be due to the fact that the number of isolates in our study was too small.

In this study, VRE was high among the age groups 43–54 (57%); this is in consistence with reports from Ethiopia, 45.5% [9]. In our study, low hemoglobin level is likely to develop VRE 19.2 times more compared to normal individuals, which is in consistence with the study conducted in Japan in which low hemoglobin level was significantly associated with the emergence of VRE [34]. However, previous antibiotic use, nutritional status, hospital admission, CD+ cell count level, ART linkage, and WHO staging were not significantly associated factors, and this is also consistent with the previous report from Gondar, Ethiopia [7].

## Figures and Tables

**Table 1:** Sociodemographic characteristics of HIV-positive and -negative clients at Dessie Referral Hospital, Northeast Ethiopia, 2017.

Sociodemographic characteristics	HIV status		Total (*n*=300), *N* (%)
	HIV positive (*n*=150), *N* (%)	HIV negative (*n*=150), *N* (%)	
*Sex*			
Male	52 (34.7)	75 (50.0)	127 (42.3)
Female	98 (65.3)	75 (50.0)	173 (57.7)

*Age group*			
18–30	44 (29.3)	95 (63.3)	139 (46.3)
31–42	52 (34.7)	47 (31.3)	99 (33.0)
43–54	41 (27.3)	7 (4.7)	48 (16)
>54	13 (4.3)	1 (0.7)	14 (4.7)

*Marital status*			
Single	25 (8.3)	66 (44)	91 (30.3)
Married	83 (55.3)	83 (55.3)	166 (55.3)
Divorced	26 (17.3)	1 (0.7)	27 (9.0)
Widowed	16 (10.7)	0	16 (5.3)

*Current*			
Rural	28 (18.7)	17 (11.3)	45 (15)

*Residence*			
Urban	122 (81.3)	133 (88.7)	255 (85)

*Education*			
Cannot read and write	19 (12.7)	5 (3.3)	24 (8.0)
Primary	55 (36.7)	34 (22.7)	89 (29.7)
Secondary	52 (34.7)	40 (26.7)	92 (30.7)
College+	24 (16.0)	71 (47.3)	95 (31.6)

**Table 2:** Antimicrobial-resistant patterns among HIV-positive and -negative clients at Dessie Referral Hospital, Northeast Ethiopia, 2017.

Resistant pattern		HIV status		Total (*n*=112), *N* (%)	*X* ^2^	*p* value
		HIV positive (*n*=85), *N* (%)	HIV negative (*n*=27), *N* (%)			
Penicillin	R	32 (37.6)	7 (25.9)	39 (34.8)	1.30	0.52
Ampicillin	R	32 (37.6)	7 (25.9)	39 (34.8)	1.30	0.52
Vancomycin	R	5 (5.9)	2 (7.4)	7 (6.3)	0.08	0.78
Gentamicin	R	38 (44.7)	4 (14.8)	42 (37.5)	8.4	0.02^*∗*^
Ciprofloxacin	R	8 (9.4)	0 (0%)	8 (7.1)	2.7	0.09
Chloramphenicol	R	7 (8.2)	3 (11.1)	10 (8.9)	0.99	0.61
Erythromycin	R	25 (29.4)	0 (0%)	25 (22.3)	11.05	0.00^*∗*^

R = resistance. ^*∗*^ = significantly associated.

**Table 3:** Profile of multidrug-resistant enterococci isolates at Dessie Referral Hospital, Northeast, Ethiopia, 2017.

Resistant pattern	Combination of antibiotics	Number (%) of isolates tested
R3	PEN, AMP, VAN, GENTA	4 (16)
	PEN, AMP, GENTA, ERY	8 (32)
	PEN, AMP, GENTA, CIP	5 (20)
	PEN, AMP, GENTA, CAF	1 (4)
	GENTA, CAF, ERY	2 (8)

R4	PEN, AMP, VAN, GENTA, ERY	3 (12)
	PEN, AMP, GENTA CAF, ERY,	1 (4)
	PEN, AMP, GENTA, CIPR, ERY	1 (4)
Total MDR	25 (100)	—

MDR = multidrug resistance; PEN = penicillin; AMP = ampicillin; GENTA = gentamicin; CIPR = ciprofloxacin; CAF = chloramphenicol; VAN = vancomycin; ERY = erythromycin; R2–R4 = number of antibiotics category resistance from 3 to 4, respectively.

**Table 4:** Antimicrobial susceptibility patterns among HIV-positive and -negative clients at Dessie Referral Hospital Northeast, Ethiopia, 2017.

Antibiotics pattern		Enterococci		Total (*n*=112), *N* (%)
		Positive (*n*=85), *N* (%)	Negative (*n*=27), *N* (%)	
Penicillin	S	51 (60)	19 (70.4)	70 (62.5)
	I	2 (2.4)	1 (3.7)	3 (2.7)
	R	32 (37.6)	7 (25.9)	39 (34.8)

Ampicillin	S	51 (60)	19 (70.4)	70 (62.5)
	I	2 (2.4)	1 (3.7)	3 (2.7)
	R	32 (37.6)	7 (25.9)	39 (34.8)

Vancomycin	S	80 (94.1)	25 (92.6)	105 (93.7)
	I	0	0	0
	R	5 (5.9)	2 (7.4)	7 (6.3)

Gentamicin	S	46 (54.1)	23 (85.2)	69 (61.6)
	I	1 (1.2)	0	1 (0.9)
	R	38 (44.7)	4 (14.8)	42 (37.5)

Ciprofloxacin	S	77 (90.6)	27 (100)	104 (92.9)
	I	0	0	0
	R	8 (9.4)	0	8 (7.1)

Chloramphenicol	S	77 (90.6)	23 (85.2)	100 (89.3)
	I	1 (1.2)	1 (3.7)	2 (1.8)
	R	7 (8.2)	3 (11.1)	10 (8.9)

Erythromycin	S	58 (68.2)	25 (92.6)	83 (74.1)
	I	2 (2.4)	2 (7.4)	4 (3.4)
	R	25 (29.4)	0	25 (22.3)

S = susceptible; I = intermediate; R = resistance.

**Table 5:** Multivariate analysis of sociodemographic characteristics and risk factors for VRE among HIV-positive and -negative clients at Dessie Referral Hospital, Northeast Ethiopia, 2017.

	Enterococci (112)		*p* value	AOR (95% CI)
	VRE (*n*=7), *N* (%)	VSE (*n*=105), *N* (%)		
*Current residence*				
Rural	4 (57.1)	15 (14.3)	0.17	5.97 (0.45–78.25)
Urban	3 (42.9)	90 (85.7)	NA	1

*Duration of antitreatment*				
<2 weeks	2 (28.6)	10 (0.9)	NA	1
≥2 weeks	4 (57.1)	19 (18.1)	0.29	0.24 (0.01–3.42)

*Low hemoglobin level*				
No	3 (42.9)	1 (0.9)	0.04	19.18 (1.08–89.36)
Yes	4 (57.1)	104 (99.0)	NA	1

VRE = vancomycin-resistant enterococci; VSE = vancomycin-susceptible enterococci; AOR = adjusted odds ratio.

## Data Availability

The data used to support the findings of this study are available from the corresponding author upon request.

## Additional Points

Species identification of enterococci was not investigated due to resource shortage and inaccessibility of testing materials. In conclusion, most of the isolates were resistant to one or more commonly used antibiotics. Prevalence of VRE and MDR enterococci in the study area was high, and this indicates that the rate of fecal colonization with VRE and MDR enterococci has increased through time, especially in HIV-positive clients than healthy individuals. The findings of this study are very crucial for indicating preferential spread of VRE and MDR in health care settings, where study subjects are readily exposed to VRE and MDR enterococci isolates; however, being HIV positive or negative was not statistically associated with the occurrence of vancomycin-resistant enterococci. Low hemoglobin level (being anemic) was significantly an associated factor for the emergence of vancomycin-resistant enterococci. Therefore, there should be a need to perform continuous surveillance, rational use of antibiotics, and more detailed study using phenotypic and genotypic methods.

## Abbreviations

AOR: Adjusted odds ratio
ART: Antiretroviral therapy
ATCC: American type culture collection
BHI: Brain-heart infusion
CD4: Cluster of differentiation 4
CDC: Centers for disease control and prevention
HI: Human immune deficiency virus
IAIs: Intra-abdominal infections
ICU: Intensive care unit
MDR: Multiple drug resistance
VAN: Vancomycin-resistant gene
VRE: Vancomycin-resistant *Enterococcus*
VRSA: Vancomycin-resistant *Staphylococcus aureus*.
